# Mechanism of baricitinib supports artificial intelligence‐predicted testing in COVID‐19 patients

**DOI:** 10.15252/emmm.202012697

**Published:** 2020-06-24

**Authors:** Justin Stebbing, Venkatesh Krishnan, Stephanie de Bono, Silvia Ottaviani, Giacomo Casalini, Peter J Richardson, Vanessa Monteil, Volker M Lauschke, Ali Mirazimi, Sonia Youhanna, Yee‐Joo Tan, Fausto Baldanti, Antonella Sarasini, Jorge A Ross Terres, Brian J Nickoloff, Richard E Higgs, Guilherme Rocha, Nicole L Byers, Douglas E Schlichting, Ajay Nirula, Anabela Cardoso, Mario Corbellino, the Sacco Baricitinib Study Group

**Affiliations:** ^1^ Department of Surgery and Cancer Imperial College London UK; ^2^ Eli Lilly and Company Indianapolis IN USA; ^3^ Luigi Sacco Department of Clinical and Biomedical Sciences University of Milan Milan Italy; ^4^ BenevolentAI London UK; ^5^ Unit of Clinical Microbiology Department of Laboratory Medicine Karolinska Institutet and Karolinska University Hospital Stockholm Sweden; ^6^ Department of Physiology and Pharmacology Karolinska Institutet and Karolinska University Hospital Stockholm Sweden; ^7^ Infectious Diseases Programme Immunology Programme Department of Microbiology and Immunology Yong Loo Lin School of Medicine National University of Singapore Singapore City Singapore; ^8^ Institute of Molecular and Cell Biology (IMCB) A*STAR (Agency for Science, Technology and Research) Singapore Singapore; ^9^ Department of Clinical, Surgical, Diagnostics and Pediatric Sciences University of Pavia Pavia Italy; ^10^ Molecular Virology Unit Fondazione IRCCS Policlinico San Matteo Pavia Italy; ^11^ Division of Infectious Diseases ASST Fatebenefratelli Sacco Milan Italy

**Keywords:** anti‐cytokine, anti‐viral, baricitinib, case series, COVID‐19, Microbiology, Virology & Host Pathogen Interaction, Pharmacology & Drug Discovery

## Abstract

Baricitinib is an oral Janus kinase (JAK)1/JAK2 inhibitor approved for the treatment of rheumatoid arthritis (RA) that was independently predicted, using artificial intelligence (AI) algorithms, to be useful for COVID‐19 infection via proposed anti‐cytokine effects and as an inhibitor of host cell viral propagation. We evaluated the *in vitro* pharmacology of baricitinib across relevant leukocyte subpopulations coupled to its in vivo pharmacokinetics and showed it inhibited signaling of cytokines implicated in COVID‐19 infection. We validated the AI‐predicted biochemical inhibitory effects of baricitinib on human numb‐associated kinase (hNAK) members measuring nanomolar affinities for AAK1, BIKE, and GAK. Inhibition of NAKs led to reduced viral infectivity with baricitinib using human primary liver spheroids. These effects occurred at exposure levels seen clinically. In a case series of patients with bilateral COVID‐19 pneumonia, baricitinib treatment was associated with clinical and radiologic recovery, a rapid decline in SARS‐CoV‐2 viral load, inflammatory markers, and IL‐6 levels. Collectively, these data support further evaluation of the anti‐cytokine and anti‐viral activity of baricitinib and support its assessment in randomized trials in hospitalized COVID‐19 patients.

## Graphical abstract

This study provides biochemical and cellular evidence confirming artificial intelligence (AI)‐predictions focused on anti‐cytokine signaling and potential anti‐viral effects for baricitinib, along with a case series, supporting its potential utility in hospitalized COVID‐19 patients.

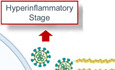

**The paper explained**

**Problem**

There are few drugs that are useful to treat COVID‐19, the largest medical crisis of this century. To try to solve this problem, using AI, we found that an oral, once‐daily medicine, baricitinib, normally used to treat adult rheumatoid arthritis (RA), may be useful in both reducing viral propagation in cells, and to mitigate the cytokine signaling seen in the hyper‐inflammatory stage of the disease. We wished to understand this further in various models and in a case series of patients.

**Results**

We found that baricitinib inhibited the signaling of cytokines we typically see as being present in hospitalized patients with COVID‐19. Using samples from a previous randomized trial in RA, we showed statistically significant declines in IL‐6 levels with baricitinib treatment. In liver spheroids designed to investigate SARS‐CoV‐2 infectivity and separately in kinase assays, we showed that baricitinib could reduce cellular infection by blockade of numb‐associated kinase members used in viral propagation. In a small case series of patients in northern Italy with bilateral COVID‐19 pneumonia, baricitinib therapy was associated with improvement in clinical, radiologic, and viral parameters along with a rapid decline in CRP and plasma IL‐6 levels.

**Impact**

Finding new drugs in our armamentarium to treat COVID‐19 would be enormously valuable. Here, we stitched together the anti‐cytokine and anti‐viral activity of baricitinib and studied it in a small number of hospitalized patients. This study represents rapid repurposing from AI to the laboratory to a potential bedside therapeutic and supports the testing of baricitinib in randomized controlled trials in COVID‐19 patients.

## Introduction

The severe acute respiratory syndrome coronavirus 2 (SARS‐CoV‐2) is currently the biggest public health challenge to the biomedical community. Despite multiple public health measures, there remains an urgent need for pharmacologic therapies to treat infected patients, minimize mortality, and optimally decrease viral shedding and subsequent transmission. Artificial intelligence (AI) allows for rapid drug development (Schneider *et al*, 2020) including repurposing existing drugs. Algorithms were used to search for approved drugs capable of inhibiting both the inflammatory damage and infectivity associated with SARS‐CoV‐2 (Richardson *et al*, 2020a; Stebbing *et al*, 2020). Baricitinib, an oral inhibitor of Janus kinase (JAK)1 and JAK2 (Fridman *et al*, 2010) approved for the treatment of moderately‐to‐severely active rheumatoid arthritis (RA) in adults, was independently hypothesized to be a therapeutic option for COVID‐19. It was considered among all molecules studied to have a unique role by virtue of its potential to both inhibit relevant cytokine signaling and have activity against the numb‐associated kinases (NAKs), AAK1 and GAK (Richardson *et al*, 2020a; Stebbing *et al*, 2020), which stimulate AP‐2‐mediated host viral propagation (Inoue *et al*, 2007; Bekerman *et al*, 2017; Owczarek *et al*, 2018).

Infection by pathogenic coronaviruses (e.g., SARS and SARS‐CoV‐2) often results in excessive cytokine and chemokine action with the development of acute respiratory distress syndrome (ARDS; Huang *et al*, 2005, 2020; Cameron *et al*, 2007; Ruan *et al*, 2020; Siddiqi & Mehra, 2020; Zhou *et al*, 2020). In patients with moderate‐to‐severe forms of these diseases, anti‐viral cytokine signaling is maintained at inappropriate levels (perhaps due to incomplete viral clearance) causing acute lung injury (Nicholls *et al*, 2003), persistent interferon (IFN) activity, and impaired T‐cell and antibody responses (Cui *et al*, 2003; Huang *et al*, 2005; Shi *et al*, 2020). In the COVID‐19 setting, ARDS is the leading cause of death and is associated with high levels of interleukin‐6 (IL‐6), which appears to be a predictor of mortality (Ruan *et al*, 2020). Therefore, treating hospitalized patients requires both improved viral clearance and restriction of the inflammatory response, with the potential to improve outcomes such as mortality and reduce admissions to intensive care units. The anti‐inflammatory benefit of baricitinib has been previously demonstrated through a reduction in a range of JAK‐STAT‐dependent cytokines (Fridman *et al*, 2010; Shi *et al*, 2014; McInnes *et al*, 2019). Phase 3 clinical trials providing safety and efficacy data have been conducted or are ongoing for baricitinib treatment in patients with autoimmune diseases including RA, atopic dermatitis, systemic lupus erythematosus, alopecia areata, juvenile idiopathic arthritis, and chronic atypical neutrophilic dermatosis with lipodystrophy and elevated temperature (CANDLE). Now, we provide biochemical and cellular evidence confirming predictions focused on anti‐cytokine signaling and potential anti‐viral effects for baricitinib, along with a case series, supporting its potential utility in hospitalized COVID‐19 patients.

## Results

### Anti‐cytokine activity for baricitinib

**Figure 1:**
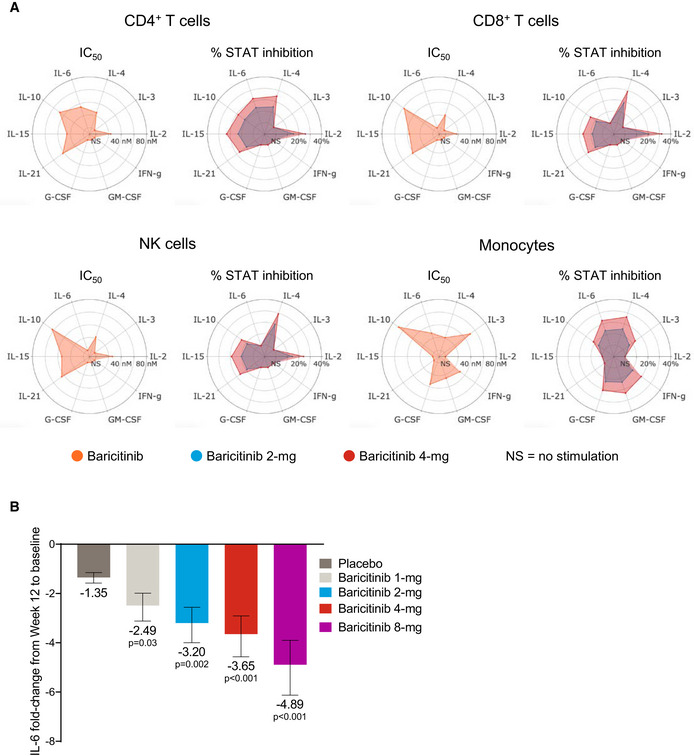
Anti‐cytokine activity of baricitinib AIC_50_ values for baricitinib (orange) in cytokine‐stimulated human CD4^+^ T cells, CD8^+^ T cells, NK cells, and monocytes are shown for IL‐2/pSTAT5, IL‐4/pSTAT6, IL‐3/pSTAT5, IL‐6/pSTAT3, IL‐10/pSTAT3, IL‐15/pSTAT5, IL‐21/pSTAT3, G‐CSF/pSTAT3, GM‐CSF/pSTAT5, and IFN‐γ/pSTAT1. Average daily percent STAT inhibition was calculated using *in vitro* concentration–response curves and published human exposure data. Cytokine treatments that did not result in sufficient pSTAT stimulation are denoted as “NS” in the radar plots.BFold change ± standard error in baseline IL‐6 plasma levels at week 12 from RA patients treated with placebo (*n* = 47), and baricitinib 1 mg (*n* = 23), 2 mg (*n* = 24), 4 mg (*n* = 23), and 8 mg (*n* = 23) in the phase 2b randomized, placebo‐controlled study NCT01469013. *P* values are for comparisons of baricitinib 1, 2, 4, and 8 mg change from baseline compared with the placebo change from baseline. Treatment effects were estimated using a mixed‐effects repeated‐measures model (SAS Proc Mixed) using log_10_‐transformed IL‐6 levels with an unstructured covariance matrix.Data information: IC_50_, half‐maximum inhibitory concentration; NK, natural killer; pSTAT, phosphorylated signal transducer and activator of transcription.

The anti‐cytokine and anti‐inflammatory activity of baricitinib was evaluated with a focus on cytokines relevant to COVID‐19 infection. We evaluated the *in vitro* pharmacology of baricitinib across relevant leukocyte subpopulations coupled to its *in vivo* pharmacokinetics to determine its effect on distinct cytokine pathways. Concentration–response curves combined with exposure data from baricitinib‐treated healthy volunteers demonstrated that baricitinib affects cytokine‐dependent phosphorylated STAT (pSTAT) inhibition to varying degrees (Fig 1A). Baricitinib inhibited signaling of cytokines implicated in COVID‐19 infection, including IL‐2, IL‐6, IL‐10, IFN‐γ, and G‐CSF, with lower half‐maximum inhibitory concentration (IC_50_) values translating to a greater overall inhibition of cytokine‐induced JAK/STAT signaling during the dosing interval (Fig 1A). Furthermore, baricitinib treatment resulted in a significant reduction (*P* < 0.05) from baseline in plasma IL‐6 at week 12 in patients with active RA who had an inadequate response to methotrexate from a phase 2b (Tanaka *et al*, 2016), randomized, placebo‐controlled, dose‐ranging study (Fig 1B).

### Anti‐viral activity for baricitinib

**Figure 2:**
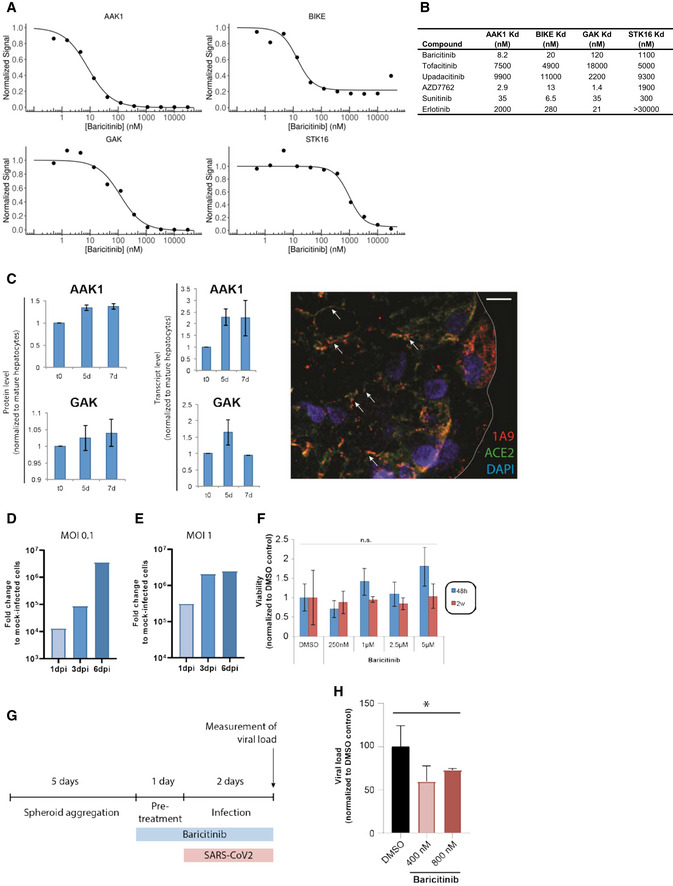
NAK‐binding affinities of baricitinib and other comparators A, BBinding affinities in equilibrium affinity constant (Kd) are shown for baricitinib (A and B), tofacitinib, upadacitinib, AZD7762 (positive control), and broad‐spectrum tyrosine kinase inhibitors sunitinib and erlotinib (B). Kd values were estimated from an 11‐point concentration–response curve with fixed top parameters and a four‐parameter logistic model (*n* = 3).CProtein and mRNA expression of relevant numb‐associated kinases in primary human liver organoids (*n* = 3) (*P* < 0.05). The immunofluorescence shows the juxtaposition of 1A9 (detecting spike protein from the SARS‐CoV‐2) in red and ACE2 in green along with the spheroid nuclei in blue as a DAPI stain (*n* = 1). Colocalization of spike and ACE2 is depicted with an arrow.D, ETime course of days (one, three, and six days) post‐infection (dpi) using liver spheroids and SARS‐CoV‐2 at 0.1 and 1.0 multiplicity of infection (MOI) (*n* = 1).FViability of spheroids was quantified by measuring intracellular ATP concentrations. Note that no significant (n.s.; Student's *t*‐test) decrease in cell viability was observed for any concentration tested (*n* = 10).GSchematic showing the timeline for the experiment shown in panel H.HViral load in infected (MOI 0.1) spheroids as measured by qPCR after treatment with 400 and 800 nM of baricitinib 48 h after infection (*P < 0.05; Student's *t*‐test). Data are mean ± standard error of mean (*n* = 3).

Next, we validated the proposed biochemical inhibitory activity of baricitinib on the NAK family members AAK1, BIKE, GAK, and STK16, some of which are hypothesized to facilitate viral propagation of coronavirus in epithelial cells (Bekerman *et al*, 2017; Owczarek *et al*, 2018; Richardson *et al*, 2020a). Baricitinib activity demonstrated affinity against AAK1 (8.2 nM), BIKE (20 nM), and GAK (120 nM; Fig 2A and B); these values are within the exposure range of the approved 2 mg (US, EU) and 4 mg (EU) once‐daily doses of baricitinib for the treatment of RA (Shi *et al*, 2014). The pharmacokinetics of baricitinib show the unbound fraction of free bioavailable drug in RA patient sera as being 326 nM area under the curve (AUC) and 652 nM (AUC) for baricitinib 2 and 4 mg, respectively (data on file). We compared the relative binding affinities of different JAK inhibitors (JAKis) for these NAKs. Notably, among JAKis approved for the treatment of RA, baricitinib uniquely demonstrated high affinity for AAK1, BIKE, and GAK, whereas tofacitinib and upadacitinib did not demonstrate high affinity for these kinases (Fig 2B). The binding affinity of baricitinib for AAK1 and GAK is similar to the binding affinity of baricitinib for JAK1 (5.9 nM) and JAK2 (5.7 nM) (Fridman *et al*, 2010).

To extend these activities on NAKs, we evaluated the effect of baricitinib in reducing viral infectivity in 3D primary human liver spheroids (Bell *et al*, 2016) infected with purified SARS‐CoV‐2 and treated with baricitinib. Specifically, we used a 3D spheroid model of primary human liver cells in which hepatocytes retain their transcriptomic, proteomic, and metabolomic phenotype and functionality for multiple weeks (Bell *et al*, 2016, 2017; Vorrink *et al*, 2017), including expression of AAK1 and GAK (Fig 2C). The overlay of the viral antigen spike (as detected with 1A9 antibody) with the ACE‐2 protein as detected by immunofluorescence, after 5 days of SARS‐CoV‐2 infection in these spheroids, provides a visual confirmation of virus infectivity in this system (Fig 2C). SARS‐CoV‐2 was able to infect human primary liver spheroids in this experimental paradigm, as demonstrated by a day post‐infection (dpi) dependent increase in viral RNA (Fig 2D and E). Baricitinib did not result in liver cell injury up to concentrations approximately eightfold higher than the clinically observed AUC values at 4 mg (AUC = 652 nM; Fig 2F). Importantly, pretreatment of spheroids with physiologically relevant concentrations of baricitinib (400 and 800 nM) significantly (*P* < 0.05) reduced viral load by 30–40%, corroborating the proposed inhibitory effects of baricitinib on AAK1 and GAK‐mediated viral propagation (Fig 2G and H). These results suggest that host cells that express these NAKs may serve as a target for baricitinib‐mediated reduction in viral propagation.

### Clinical case series using baricitinib

**Figure 3:**
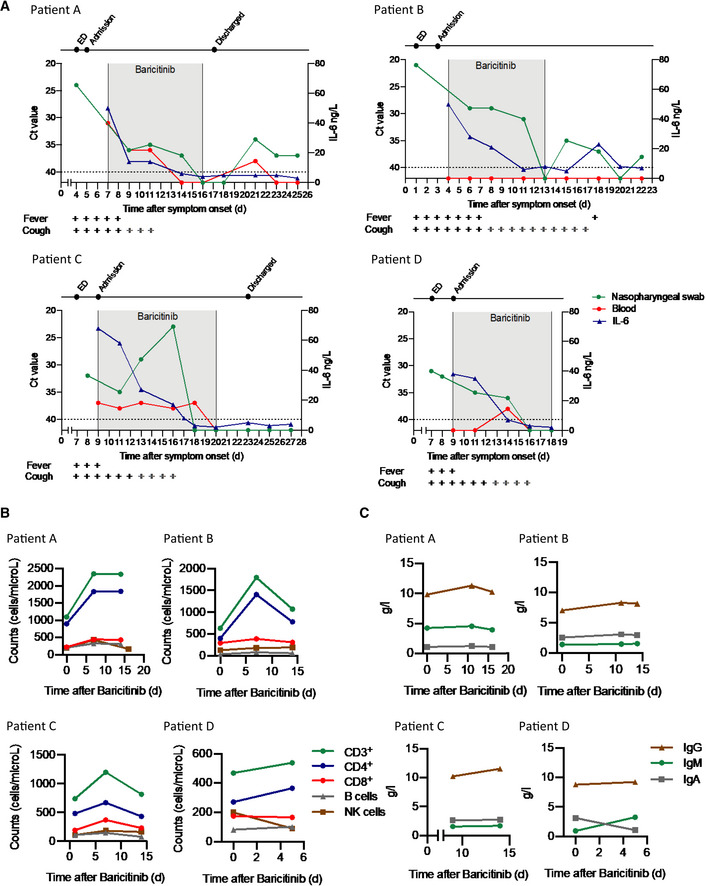
Viral detection and immunological features of four COVID‐19 patients treated with baricitinib ADetection of SARS‐CoV‐2 in nasopharyngeal swabs and peripheral blood using the GeneFinder™ COVID‐19 Plus RealAmp Kit assay (ELITechGroup S.p.A., Turin, Italy). Three viral target genes, RdRp, N, and E, together with the housekeeping gene GAPDH, were simultaneously amplified. Here, the most sensitive target gene, N, is shown. Samples with Ct values > 40 were defined as negative. A dashed horizontal line indicates the cutoff. IL‐6 values are shown in the same graph. Time of baricitinib treatment is highlighted (Patients A, B, and D for 10 days, Patient C for 12 days) with total time of evaluation on the *x*‐axis. Fever and cough symptoms are indicated. The bars in gray indicate that cough has improved but has not resolved.BLevels of CD3^+^, CD4^+^, CD8^+^, B cells (CD19^+^), and NK cells (CD3^−^, CD16^+^, CD56^+^) were determined using the AQUIOS CL Flow *Cytometry* System (Beckman Coulter).CTotal serum IgG, IgA, and IgM levels are shown for all patients.Data information: Ct, cycle threshold; E, envelope membrane; ED, emergency department; N, nucleocapsid protein; NK, natural killer; RdRp, RNA‐dependent RNA polymerase.

Following the recent publications by Richardson *et al* (2020a) and Stebbing *et al* (2020), COVID‐19 patients were treated with baricitinib in a pilot study in Milan, Italy. Four patients with bilateral COVID‐19 pneumonia, who presented with varying degrees of disease severity (Table 1), were included in this pilot study; three individuals (Patients B, C, and D) were clinically unstable with moderate‐to‐severe disease. All four patients were admitted to the ward from the emergency department in March 2020. As shown in Table 1, Patient A was a female nurse, aged 29. Patient B, a 76‐year‐old male, had significant co‐morbidities (a former smoker, arterial hypertension (AH), chronic obstructive pulmonary disease [COPD], coronary artery disease (CAD), and had undergone an aortic aneurysm Endurant II graft repair on February 25, 2020). Patient C, a 57‐year‐old male, had co‐morbidities including AH and COPD (non‐smoker); he suddenly deteriorated a few hours following hospitalization, and was placed on continuous positive airway pressure at the time baricitinib was initiated. Patient D, a 51‐year‐old male, had a high body mass index (BMI) of 35. All four patients had detectable plasma IL‐6 levels (Fig 3A) with markedly raised inflammatory markers (C‐reactive protein [CRP]), as would be expected in patients with COVID‐19 pneumonia.

**Table 1:** Clinical characteristics of patients in baricitinib COVID‐19 case study

	Patient A	Patient B	Patient C	Patient D
Age (years)	29	76	57	51
Gender	Female	Male	Male	Male
Coexisting conditions	None	AH, COPD, CAD	AH, COPD	Obesity
BMI	22	28	29	35
Medications	Combined oral contraception	Losartan, propranolol, omeprazole, aspirin, LMW heparin	Losartan, hydrochlorothiazide, inhaled beclomethasone	None
Smoking	No	Former	No	No
Data at presentation				
Symptoms	Fever, dry cough, and myalgias	Fever, dry cough	Fever, dry cough, dyspnea	Fever, dry cough, headache
Blood oxygen levels PaO2 (mmHg)	111	81	62	80
Alveolar oxygen gradient (KPa)	0	4.7	12.9	4.9
SpO2 (%)	98	97	94	91
RR/min	26	32	28	28
Baricitinib dose/days on treatment	4 mg for 10 days	2 mg for 10 days	4 mg for 12 days	4 mg for 10 days
Antibiotic therapy	None	Ceftriaxone, tazocin, fosfomycin	Ceftriaxone, azithromycin	None

Study note: According to the eligibility criteria, Patient A tested negative for pregnancy and all four patients tested negative for HIV‐1/2, and tuberculosis using QuantiFERON‐TB Gold Plus. AH, arterial hypertension; BMI, body mass index; CAD, coronary artery disease; COPD, chronic obstructive pulmonary disease; RR, respiratory rate.

As shown in Fig 3A, all four patients showed improvement with baricitinib treatment in signs and symptoms such as cough, fever, and reduction in plasma IL‐6 levels, along with a reduction in the SARS‐CoV‐2 RNA viral load, as detected by the real‐time reverse transcriptase–polymerase chain reaction (RT–PCR) signal from the nasopharyngeal carriage. Stringent criteria were used for RNA detection of SARS‐CoV‐2 in nasopharyngeal carriage and peripheral blood. Real‐time RT–PCR was performed on three distinct viral gene targets (Corman *et al*, 2020; Appendix Table S1), using the most sensitive target (N gene) and a cutoff using Ct values > 40 for the analyses illustrated in Fig 3A, in contrast to others, for example in the hydroxychloroquine study (Ct values ≥ 35; Gautret *et al*, 2020). Only two of the patients (Patients A and C) had detectable viral RNA in their peripheral blood (Fig 3A and Appendix Table S1).

**Figure 4:**
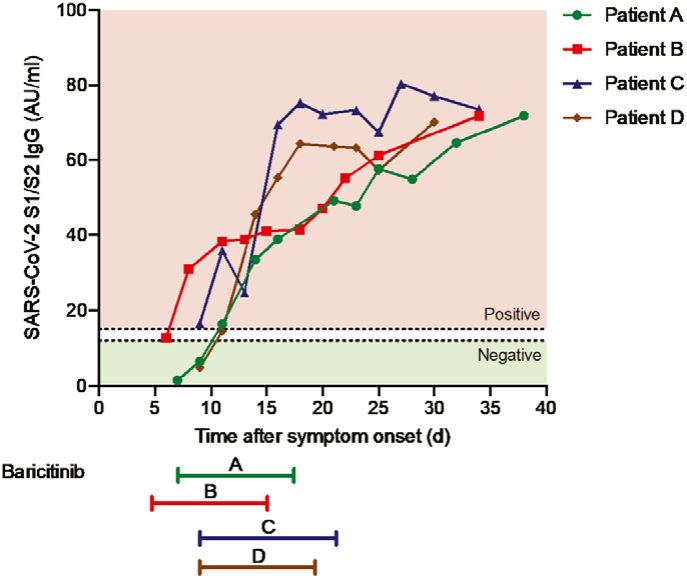
Sero‐conversion of patients during baricitinib treatment Detection of IgG antibodies against the S1/S2 antigens of SARS‐CoV‐2. Time of baricitinib treatment is indicated for all patients (Patients A, B, and D for 10 days, Patient C for 12 days).

**Figure 5:**
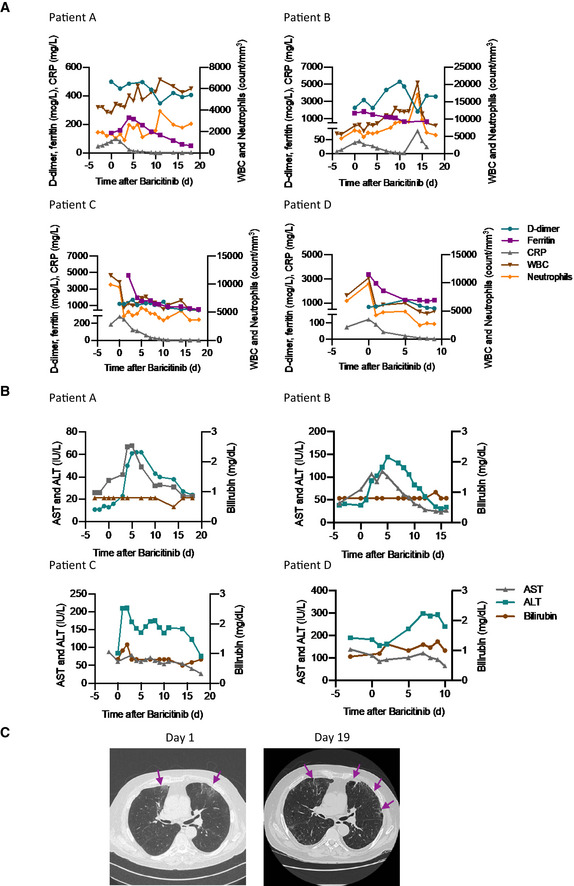
Standard laboratory and radiologic features of the four COVID‐19 cases treated with baricitinib ALevels of D‐dimer, CRP, ferritin, white blood cells, and neutrophils are shown for all patients.BLevels of aspartate aminotransferase, alanine aminotransferase, and bilirubin are shown for all patients.CChest CT scan for Patient C on day 1 and day 19 from symptom onset showing clinical improvement over time. Day 1 CT scan shows ground‐glass opacity (arrows) sub‐pleurally in the lower lobes bilaterally (early stage one according to Pan *et al*, 2020). Day 19 CT scan shows that consolidation was gradually absorbed with evident residual fibrosis and emphysema bubbles (arrows) in the site of the early lesions (absorption stage 4 according to Pan *et al*, 2020).Data information: ALT, alanine aminotransferase; AST, aspartate aminotransferase; CRP, C‐reactive protein; CT, computed tomography; WBC, white blood cells.

There were nominal changes in lymphocyte counts throughout the course of treatment with baricitinib in all four patients (Fig 3B). In addition, changes in IgG and IgM (Fig 3C) suggest adequate levels consistent with the confirmed sero‐conversion for 3/4 of the patients after initiation of baricitinib (Fig 4). Most importantly, all four patients achieved sero‐conversion as evidenced by the presence of neutralizing antibodies against the S1 and S2 spike proteins of SARS‐CoV‐2 after baricitinib exposure (Fig 4). Neutrophil and total white blood cell counts tracked with improvement in disease severity (Fig 5A). In addition, all four patients demonstrated improvement in their CRP, ferritin, and D‐dimer levels (Fig 5A). Notably, Patient C, who had the lowest PaO_2_ at baseline, showed radiographic improvement in lymphocytic infiltrates when comparing the computer tomography scan from day 1 to day 19 (Fig 5C). Overall, a total of 10 days of dosing for Patients A, B, and D and 12 days for Patient C, with baricitinib 4 mg once‐daily orally in Patients A, C, and D, and 2 mg in Patient B (in accordance with the label guidelines for treatment of RA), was sufficient to document in all patients improved lung function, resolution of their illness, and reductions in viral load, plasma IL‐6, ferritin, and CRP levels. Baricitinib treatment was associated with a rapid and consistent improvement in clinical, radiologic, virologic, inflammatory, and cytokine measures in this diverse group of patients, including 3 (Patients B, C, and D) who were deemed to be moderately‐to‐severely unwell and at high risk of deteriorating rapidly (i.e., those apart from the 29‐year‐old nurse). Transient increases in liver aminotransferases were observed in all four patients, with no changes in other liver enzymes or bilirubin (Fig 5B); however, these liver enzyme elevations improved within 72 h without interrupting baricitinib treatment, suggesting that these elevations may not be causally related to baricitinib treatment, but may be reflective of disease severity (Fig 5B). In general, these data demonstrate that treatment with baricitinib in these four patients with bilateral COVID‐19 pneumonia was well‐tolerated. Collectively, this limited case series provides preliminary evidence that baricitinib treatment may lower inflammatory burden and may result in a reduction in disease severity in COVID‐19 patients.

## Discussion

The pharmaceutical interventions for the treatment of COVID‐19 patients proposed to date include testing anti‐viral mechanisms, either in combination or alone, along with anti‐malarials and immunomodulators (Siddiqi & Mehra, 2020). One such potential pharmacological approach is the use of immunomodulators in the subset of patients who develop a cytokine storm associated with pulmonary involvement including ARDS that leads to a rapid deterioration of the co‐morbid conditions in such patients (Appendix Fig S1; Huang *et al*, 2020; Siddiqi & Mehra, 2020; Zhou *et al*, 2020). In this report, we demonstrate that baricitinib is an inhibitor of cytokines implicated in intensive care unit‐bound COVID‐19 patients, which have been collated from several reports, including IL‐2, IL‐6, IL‐10, IFN‐γ, and G‐CSF (Huang *et al*, 2020; Ruan *et al*, 2020; Zhou *et al*, 2020). Elevated IL‐6 and hyperferritinemia were predictors of death in COVID‐19 patients in China (Ruan *et al*, 2020; Zhou *et al*, 2020). The relevance of IL‐6 in COVID‐19‐associated ARDS was recently shown in an open‐label study where blockade with tocilizumab, an IL‐6R antibody, resulted in a rapid recovery of peripheral oxygen saturation and recovery from febrile signs and symptoms (Xu *et al*, 2020). Baricitinib has been previously shown to inhibit IL‐6‐induced MCP‐1 production from human peripheral blood mononuclear cells (Fridman *et al*, 2010). In addition to a marked and rapid reduction in levels of human serum CRP in adult RA patients previously described (Tanaka *et al*, 2016), baricitinib treatment also reduced the mean change from baseline of plasma IL‐6 in adult RA patients. The anti‐inflammatory effects of baricitinib have also been demonstrated by the reduction in serum levels of IFN‐γ, IP‐10, GM‐CSF, and MCP‐1 in pediatric patients with steroid‐dependent chronic inflammation, resulting in control of disease activity and the ability to wean or taper steroids (Sanchez *et al*, 2018).

Another pharmacologic strategy could include targeting members of the NAK family (AAK1, GAK, BIKE, and STK16) that activate the AP‐2 scaffolding protein vital to viral entry and propagation (Bekerman *et al*, 2017; Owczarek *et al*, 2018). Previous reports demonstrated inhibiting such kinases was effective in reducing cellular infectivity for viruses that rely on the activity of AP‐2 such as HCV, dengue, HIV, SARS, and Ebola (Chaudhuri *et al*, 2007; Neveu *et al*, 2015; Bekerman *et al*, 2017; Owczarek *et al*, 2018). While baricitinib demonstrated potent inhibition of several NAKs that phosphorylate the AP‐2 adaptor and reduced viral infectivity in primary human liver spheroids, it is still unknown whether this inhibition inhibits SARS‐CoV‐2 propagation in COVID‐19‐infected patients.

The aforementioned four COVID‐19 patients offer support to the mechanism of action of baricitinib as an anti‐cytokine and a potential anti‐viral agent. Reductions in inflammatory markers of COVID‐19 diseases, such as IL‐6, CRP, and ferritin, are consistent with expected anti‐inflammatory activity. While the consistent reduction in viral RNA observed in all four patients (measured from their nasopharyngeal carriage) is suggestive of reduction in viral load, this could be a consequence of the natural ability in these patients to clear the virus. At the time of manuscript submission, a few weeks have elapsed since cessation of baricitinib. Active clinical, biochemical, and virologic surveillance of each patient to monitor any sequelae, including the possible appearance of an immune reconstitution inflammatory syndrome or molecular evidence of *de novo* SARS‐CoV‐2 replication in the respiratory tract or peripheral blood, clearly shows that all four patients were devoid of viral load and signs and symptoms of COVID‐19.

Given many unknowns concerning this novel SARS‐CoV‐2 virus, it is necessary to consider safety concerns alongside potential efficacy. As an effective immunomodulatory agent used for the treatment of RA, baricitinib was associated with an increased risk of infections such as upper respiratory tract infections and herpes zoster (Eli Lilly and Company, 2019). Baricitinib treatment in RA patients has been associated with increased incidence of deep vein thrombosis (DVT), and it is recommended that appropriate prophylactic measures be considered with use of baricitinib in hospitalized patients with higher risk of DVT (Eli Lilly and Company, 2019). This is especially relevant given recent findings of thrombotic complications in COVID‐19 patients (Klok *et al*, 2020). As baricitinib is an inhibitor of IFN‐responsive genes (Sanchez *et al*, 2018), its potential impact on the subsequent development of protective humoral and cell‐mediated anti‐viral immunity needs to be assessed in the context of COVID‐19 infection. Of relevance, RA patients on long‐term baricitinib treatment achieved satisfactory humoral responses to pneumococcal conjugate (PCV‐13) vaccination suggesting that humoral responses remain partially intact (Winthrop *et al*, 2019). Changes seen in lymphocyte subsets in this limited clinical case series suggest that baricitinib treatment does not reduce T‐cell subsets (CD3^+^ and CD4^+^) or NK cells. It is too early to ascribe observed reductions in SARS‐CoV‐2 RNA loads in the nasopharyngeal carriage to a putative anti‐viral activity of baricitinib, since the underlying T‐cell subset levels were maintained in these treated patients. This is consistent with previous reports wherein various T‐cell and B‐cell subsets remained within normal reference ranges after initiation of baricitinib treatment in RA patients (Tanaka *et al*, 2018). It is, however, reassuring to underscore that after treatment with an immunomodulatory agent such as baricitinib, we could measure IgG anti‐S1‐ and anti‐S2‐neutralizing antibodies. Most importantly, three out of four patients, while under baricitinib exposure, achieved sero‐conversion, as evidenced by the appearance of IgG antibodies with neutralizing activity against SARS‐CoV‐2. Moreover, in all four patients, a progressive rise in the titer of these neutralizing antibodies was observed during follow‐up. However, the frequency and time course of this occurrence would require prospective validation in randomized controlled trials (Ottoviani & Stebbing, 2020). Translational research from these studies will also help delineate the relative contributions of the humoral, cellular, and innate immune responses to SARS‐CoV‐2.

Baricitinib has a well‐established safety profile in patients with RA over long‐term treatment; in 10,127 patients‐year of exposure and up to 6.9 years of duration, the incidence rate of serious infection was 2.8/100 patient‐years and stable over time (Genovese *et al*, 2019). Furthermore, in 89 individuals treated with baricitinib in four separate single‐center studies across northern Italy, no over‐arching adverse safety signals have been observed with transient use (< 14 days) of baricitinib in COVID‐19 patients (Francesco Menichetti, Fabio Lena, Fabrizio Cantini, and Pinuccia Omodeo, *personal communications*). Vigilance for detecting serious adverse events and appropriate management of potential complications is essential given the inhibition on IFN‐responsive genes by baricitinib (Sanchez *et al*, 2018). Therefore, the impact of baricitinib on the subsequent development of protective humoral and cell‐mediated anti‐viral immunity in COVID‐19 patients must be evaluated in randomized clinical trials (Ottoviani & Stebbing, 2020). Importantly, baricitinib is administered orally once a day and has a low cytochrome P_450_ inhibitory activity with a low drug–drug interaction risk; its short half‐life (approximately 12 h in RA patients) and renal elimination as the main clearance mechanism make it fast to wash‐out if necessary (Eli Lilly and Company, 2019).

Collectively, these data provide preliminary evidence that baricitinib could be tested as an effective intervention strategy to stem the cytokine storm and viral propagation seen in hospitalized COVID‐19 patients. The finding that baricitinib is a potent AAK1/BIKE/GAK inhibitor that may reduce host cell infectivity, along with reaffirmation of its anti‐cytokine profile, provides reasons to study this intervention in randomized clinical trials. We cannot over‐interpret the findings from the case series described in this manuscript. Therefore, results from such randomized trials, notably the adaptive National Institute of Allergy and Infectious disease (ADAPTIVE COVID‐19 ACCT2), will be central to define efficacy and safety of baricitinib and to manage effective clinical care as this outbreak continues to expand across the globe.

## Materials and Methods

### Leukocyte preparation and experimental design

Leukocyte preparation and experimental design were largely performed as previously described (McInnes *et al*, 2019). Whole blood samples from healthy donors (*N* = 6) were apheresed, and leukocyte‐enriched fractions were transferred to the company Primity Bio (Fremont, CA, USA). Immediately following apheresis, approximately 600,000 cells were plated in 100 μl into 96‐well plates and incubated with baricitinib using an 8‐point dose range from 0.1 nM to 10,000 nM for 1 h prior to stimulation with cytokines for 15 min at 37°C. Baricitinib (Eli Lilly and Company) was prepared as 10 mM stocks in dimethyl sulfoxide. Cytokines were used at the following concentrations: IL‐2 (4 ng/ml), IL‐3 (12 ng/ml), IL‐4 (350 pg/ml), IL‐6 (1.5 ng/ml), IL‐10 (12 ng/ml), IL‐15 (850 pg/ml), IL‐21 (200 pg/ml), IFN‐γ (100 pg/ml), G‐CSF (5 ng/ml), and GM‐CSF (5 pg/ml). The choice of cytokine concentrations and incubation conditions was optimized in order to ensure consistent signaling in alternate cell types and pSTAT readouts. After stimulation, cells were fixed, permeabilized, and fluorescence‐barcoded, and multicolor flow cytometry was performed as previously described to quantify STAT phosphorylation in gated leukocyte subpopulations (McInnes *et al*, 2019). For a given case (stimulation, cell type, and pSTAT combination), the IC_50_ was determined if there was a consistent response to the stimulus as described in the statistical analysis section. The primary pSTAT observed for each stimulus is reported. Leukocyte populations were defined as CD20^+^ (B cells), CD3^+^CD4^+^ (CD4^+^ T cells), CD3^+^CD4^−^ (CD8^+^ T cells), and CD3^−^CD56^+^ (natural killer cells), and by forward and side scatter (monocytes). Whole blood samples from healthy volunteers were obtained under a protocol approved by the Institutional Review Board of Stanford University (reference numbers eProtocol #13942 and IRB Registration #5136). The IRB provided a Waiver of Consent because leukocyte‐enriched fractions are by‐products of the blood donation.

### IL‐6 changes in baricitinib‐treated RA patients

Patient samples were obtained from the double‐blind, randomized, placebo‐controlled, phase 2b study NCT01469013. Patients had moderate‐to‐severe active adult‐onset RA, despite stable methotrexate treatment. Patients (*N* = 145) were randomized (2:1:1:1:1) to placebo or once‐daily oral 1, 2, 4, or 8 mg baricitinib for 12 weeks (Tanaka *et al*, 2016). Plasma IL‐6 levels were analyzed using an enzyme immunoassay. Treatment effects were estimated using a mixed‐effects repeated‐measures model (SAS Proc Mixed) using log10‐transformed IL‐6 levels with an unstructured covariance matrix. NCT01469013 was conducted in accordance with ethical principles of the Declaration of Helsinki and Good Clinical Practice guidelines. All investigation sites received approval from the appropriate authorized institutional review board or ethics committee. All patients provided written informed consent before the study‐related procedures were undertaken.

### Human NAK assays and viral infection

AAK1‐, BIKE‐, GAK‐, and STK16‐binding assays were performed with DNA‐tagged recombinant human proteins derived from HEK‐293 cells or *Escherichia coli*. AAK1 kinase‐tagged T7 phage strains were prepared in an *E. coli* host derived from the BL21 strain. *E. coli* were grown to log phase and infected with T7 phage and incubated with shaking at 32°C until lysis. The lysates were centrifuged and filtered to remove cell debris. The remaining kinases (BIKE, GAK, and STK16) were produced in HEK‐293 cells and subsequently tagged with DNA for qPCR detection. Streptavidin‐coated magnetic beads were treated with biotinylated small molecule ligands for 30 min at room temperature to generate affinity resins for kinase assays. The liganded beads were blocked with excess biotin and washed with blocking buffer (SeaBlock; Thermo Scientific Pierce, 1% BSA, 0.05% Tween 20, 1 mM DTT) to remove unbound ligand and to reduce non‐specific binding. Binding reactions were assembled by combining kinases, liganded affinity beads, and test compounds in 1× binding buffer (20% SeaBlock, 0.17× PBS, 0.05% Tween 20, 6 mM DTT). Test compounds were prepared as 111× stocks in 100% DMSO. Equilibrium affinity constants (Kds) were determined using an 11‐point threefold compound dilution series with three DMSO control points.

All compounds for Kd measurements were distributed by acoustic transfer (non‐contact dispensing) in 100% DMSO. The compounds were then diluted directly into the assays such that the final concentration of DMSO was 0.9%. All reactions were performed in polypropylene 384‐well plates with a final volume of 0.02 ml. The assay plates were incubated at room temperature with shaking for 1 h, and the affinity beads were washed with wash buffer (1× PBS, 0.05% Tween 20). The beads were then re‐suspended in elution buffer (1× PBS, 0.05% Tween 20, 0.5 μM non‐biotinylated affinity ligand) and incubated at room temperature with shaking for 30 min. The kinase concentration in the eluates was measured by qPCR.

### SARS‐CoV‐2 infection of human liver spheroids

Protein levels of AAK1 and GAK were pulled out from tandem mass tag (tmt)‐based proteomic datasets (Oliva‐Vilarnau *et al*, In press) generated from total protein of 192 spheroids at the Clinical Proteomics Mass Spectrometry facility (Science for Life Laboratory, Stockholm, Sweden). Both proteins had a protein spectrum match (PSM) level of > 1 in all analyzed samples. The corresponding RNA expression values were obtained by bulk RNA sequencing (poly‐A) of a minimum of 100 ng total RNA at the National Genomics Infrastructure (NGI) facility (Science for Life Laboratory, Stockholm, Sweden).

SARS‐CoV‐2 (GenBank accession number MT093571) was isolated from a nasopharyngeal sample of a patient in Sweden on Vero E6 cells. Cryopreserved PHH (BioIVT, USA) was thawed and seeded into 96‐well ultra‐low attachment plates (Corning) with 1,500 cells per well as previously described (Bell *et al*, 2016). Spheroids were pre‐exposed to baricitinib for 24 h starting five days after cell seeding. At day 6 of culture, cells were exposed to SARS‐CoV‐2 at a multiplicity of infection of 0.1 in triplicate for 48 h. After 48 h, spheroids were washed with PBS, pooled (32 wells/condition), and lysed using TRIzolTM (Thermo Fisher). RNA was extracted using Direct‐zol Mini Kit (Zymo Research), and relative level of viral RNA was determined by qRT–PCR as previously described (Monteil *et al*, 2020).

### Immunofluorescence microscopy

Primary human hepatocyte spheroid cryo‐sections were washed twice with PBS for 10 min at room temperature. Afterward, samples were blocked with PBTA buffer (5% BSA, 0.25% Triton X‐100, 0.01% NaN_3_ in PBS) for 2 h at room temperature prior to an overnight incubation at 4°C with the monoclonal primary antibody anti‐1A9 (diluted in PBTA to a final concentration 5 μg/ml). Samples were washed 3 × 15 min with PBS at room temperature before incubation with the secondary antibody (donkey anti‐mouse diluted in PBTA 1:500) for 2 h at room temperature. Subsequently, samples were washed 3 × 15 min with PBS at room temperature and mounted with DAPI Gold Antifade.

### Clinical case series

#### Study design and participants

On the basis of the two reports of AI‐derived discovery of baricitinib in COVID‐19 (Richardson *et al*, 2020a; Stebbing *et al*, 2020), the Sacco Baricitinib Study Group and Imperial College developed a protocol to assess baricitinib in a small case series of patients, acknowledging potential risks of pulmonary infections (Favalli *et al*, 2020; Richardson *et al*, 2020b) based on its mechanism of action while leveraging the hypothesis for a potential therapeutic benefit in COVID‐19. Herein, we describe the cases from the independent research conducted. Ethics and local Institutional Review Board approval was authorized on March 16, 2020 (number 14581/2020), which provided permission to treat three patients with bilateral COVID‐19 pneumonia with baricitinib. Three patients (Patients A, B, and C) were immediately recruited that day, having been admitted to the wards from the emergency department. A supplementary approval was sought on March 25, 2020, and obtained to treat a fourth patient (Patient D) for validation purposes.

Inclusion criteria for compassionate use of an investigational oral medicine (albeit one that was approved in another indication) for patients with signs of severe illness at diagnosis or secondary clinical aggravation (respiratory symptoms or general signs) were based on World Health Organization (WHO) criteria for severe pneumonia caused by SARS‐CoV‐2 (WHO, 2020a).

Written informed consent was obtained from each patient, and 4 mg oral baricitinib was administered once‐daily according to the label for treatment of RA with a 2 mg lower dose as per the label, for 10–12 days.

Criteria for patient discharge with recovery were from the European Centre for Disease Prevention and Control guidelines (ECDC, 2020). The open‐access Clinical Characterization Protocol for Severe Emerging Infections of the International Severe Acute Respiratory and Emerging Infection Consortium (supported by WHO) has been updated and used in response to COVID‐19 (Dunning *et al*, 2014).

#### Procedures

Clinical samples for SARS‐CoV‐2 diagnostic testing were obtained according to WHO guidelines (WHO, 2020b). For each patient, a sampling strategy was implemented in which nasopharyngeal and blood samples were obtained regularly from hospital admission, and subsequently once every two or three days until patient discharge. At the time of manuscript submission, these four patients continue to be monitored for COVID‐19 disease. Upper respiratory samples were nasopharyngeal swabs, and blood samples were EDTA tubes adapted for RT–PCR. All samples were analyzed in the same center as the patients where procedures for RNA extraction, real‐time RT–PCR (rRT–PCR), were undertaken.

Throat swabs and plasma for each patient were processed using the automated ELITe InGenius^®^ system and the GeneFinderTM COVID‐19 Plus RealAmp Kit assay (ELITechGroup, France). The reaction mix was manually prepared (according to the manufacturer's instruction) and loaded onto the system with other reagents, while RNA was extracted from 200 μl of sample and eluted in 100 μl; the final reaction volume consisted of 5 μl of RNA plus 15 μl of reagent mix. The RT–PCR setup according to the manufacturer's instructions was 50°C for 20 min, 95°C for five minutes plus 45 cycles at 95°C for 15 s, and 58°C for 60 s. Three target genes, RNA‐dependent RNA polymerase (RdRP), nucleocapsid protein (N), and envelope membrane protein (E) were simultaneously amplified and tested. A cycle threshold value (Ct value) < 40 was defined as a positive test result, and a Ct value greater than 40 was defined as a negative outcome according to our criteria. The quality of nasopharyngeal swabs was checked using the CELL Control r‐gene Kit (bioMérieux).

### Primer and probe sequences used for SARS‐CoV‐2 rRT–PCR

**Table:** 

Assay	Oligonucleotide	Sequence
N	Forward primer (HKU‐NF)	TAATCAGACAAGGAACTGATTA
N	Reverse primer (HKU‐NR)	CGAAGGTGTGACTTCCATG
N	Probe (HKU‐NP)	FAM‐GCAAATTGTGCAATTTGCGG‐TAMRA
E	Primer E_Sarbeco_F1	ACAGGTACGTTAATAGTTAATAGCGT
E	Primer E_Sarbeco_R2	ATATTGCAGCAGTACGCACACA
E	Probe E_Sarbeco_P1	FAM‐ACACTAGCCATCCTTACTGCGCTTCG‐BBQ
RdRP	Primer RdRP_SARSr‐F2	GTGARATGGTCATGTGTGGCGG
RdRP	Primer RdRP_SARSr‐R1	CARATGTTAAASACACTATTAGCATA
RdRP	Probe RdRP_SARSr‐P1	FAM‐CCAGGTGGWACRTCATCMGGTGATGC‐BBQ
RdRP	Probe RdRP_SARSr‐P2	FAM‐CAGGTGGAACCTCATCAGGAGATGC‐BBQ

Primer and probe sequences for gene N are from the Hong Kong protocol (HKUMed, 2020). Primer and probe sequences for genes E and RdRp are from the Berlin protocol (Drosten *et al*, 2020). E, envelope membrane; N, nucleocapsid protein; RdRp, RNA‐dependent RNA polymerase; rRT–PCR, real‐time reverse transcriptase–polymerase chain reaction.

IL‐6 levels were determined on the fully automated immunochemistry platform COBAS e601 (Roche Diagnostics) by the proprietary electrochemiluminescent immunoassay (ref. 05109442190, lot 43676101) using 30 μl of serum. SARS‐CoV‐2 serum IgG and serum IgM were qualitatively assessed using the immunochromatographic COVID‐19 IgG/IgM Rapid Test, following instructions (PRIMA Lab SA, Balerna, Switzerland). Briefly, 10 μl of EDTA‐anticoagulated plasma was loaded into the well of each cassette. 80 μl of buffer was subsequently added to the well. The presence of SARS‐CoV‐2 IgG and IgM was visually determined after an incubation of 10 min.

Immunoglobulin levels (IgG, IgA, IgM) were measured on the automated platform AU 480 (Beckman Coulter) by their respective proprietary immunoturbidimetric assays; complete blood cell count was performed on XN‐9000 Automated Hematology System (Sysmex); coagulation tests including D‐dimer levels were carried out on the fully automated hemostasis testing analyzer ACL 750 TOP (Werfen) using proprietary reagents; alanine aminotransferase and aspartate aminotransferase activity, CRP, and serum ferritin concentrations were determined on the fully automated platform for Clinical Chemistry and Immunoassay Alinity ci (Abbott Diagnostics) with the proprietary reagents. For measuring AST and ALT, the assays with the addition of pyridoxal‐5‐phosphate (P‐5′‐P) traceable to the reference measurement system, for CRP the high‐sensitive immunoturbidimetric assay, and for ferritin levels the chemiluminescent microparticle immunoassay, were respectively used.

On the wards, standard laboratory and clinical management according to two treating physicians’ discretions (MC and GC) were used. The chest CT shown was performed using a single inspiratory phase in one commercial multidetector CT scanner (General Electric Healthcare Revolution64) with a breath‐holding protocol (tube voltage 120 kVp, thickness 1.4 mm, increment of 1.4 mm, mean CTDIvol 19 mGy).

### Sero‐conversion assay

IgG antibodies against the S1/S2 antigens of SARS‐CoV‐2 using the LIAISON SARS‐CoV‐2 S1/S2 IgG Kit (DiaSorin S.p.A., Saluggia, Italy) were detected. The IgG antibody concentrations are expressed as arbitrary units (AU/ml) with values < 12 being interpreted as negative; between 12 and 15 as equivocal; and > 15 as positive. Dashed horizontal lines indicate the cutoff values.

### Statistical analysis

#### NAK‐binding assays

The concentration–response curve (CRC) fitting for NAK‐binding assay as previously described was normalized by dividing by the mean of three replicates of the 0 nM treatment condition. Four‐parameter logistic (4PL) curves were fit with the normalized assay signal as the response and log_10_ compound concentration as the independent variable using the R drc package (R version 3.6.0 and drc version 3.0). The curve top parameter was fixed to a value of one, while all other parameters were estimated in the curve fitting. Kd values were determined using the absolute half‐maximum efficacy concentration (EC_50_) values estimated from the logistic model.

#### IC_50_ values for cytokine signaling

IC_50_ values were determined by analyzing the mean fluorescence intensity (MFI) of cytokine‐stimulated samples in the presence of the designated concentration of compound. For a given case (stimulation, cell type, and pSTAT combination), the MFI for unstimulated and stimulated cells was determined for each donor. To ensure that a biologically relevant signal was induced, CRCs were only analyzed when a consistent response to stimulus was observed as described below. Data for baricitinib were analyzed with a mixed‐effects model having compound as a fixed effect and donor as a random effect.

#### Selection of cases for analysis, fitting, and selection of CRC curves for anti‐cytokine activity of baricitinib

Two sets of criteria for reporting an IC_50_ value and computing a steady‐state activity (SSACTIVITY) curve were used: one at the case level and another at the individual curve level. At the case level, the median across the six donors of the minimum stimulated to unstimulated ratio over two replicates is computed, and it is required that the median minimum ratio be above 1.5. Out of a total of 85 cases, 43 were selected according to these criteria. Once a case met this criterion, 4PL curves were fit to the curve response concentration data. If an individual curve had top outside of the 80–120% activity range or bottom outside of the (−20, 20%) range, the 4PL was refitted with these constraints. After a CRC was fit, the following quality criteria were evaluated: (i) R2 above 0.8; (ii) SE of ln(I IC_50_) below 10; and (iii) estimated IC_50_ is within a fivefold difference in the minimum and maximum experimented concentrations. A curve failing any of these criteria was discarded.

#### Estimation of daily percent inhibition

The individual 4PL CRCs were combined with population pharmacokinetic (PK) curves to calculate the average steady‐state daily percent inhibition. The PK profiles of baricitinib were estimated from a two‐compartment model with zero‐order absorption that was developed using data from healthy volunteers from three phase 1 clinical trials with once‐daily 2–20 mg or twice‐daily 5 mg. Protein‐binding effects were accounted for by replacing the *in vitro* IC_50_ with an adjusted IC_50_ value computed by dividing the IC_50_ value for each donor by the proportion of compound unbound. Protein‐bound adjusted CRCs were constructed by replacing the *in vitro* IC_50_ value with the adjusted value. The average daily percent inhibition for a subject was obtained by entering the steady‐state PK concentrations into the adjusted CRCs, computing the area under this curve, and dividing it by 24 h. Using the individual donor values for average daily percent inhibition, a mixed‐effects model having compound as a fixed effect and donor as a random effect was fit. The reported estimates for population average SSACTIVITY are taken as the least‐squares means from this model. No transformations were undertaken to keep the estimates of SSACTIVITY within the 0–100% range.

## Author contributions

VK wrote the first draft of the manuscript. VK, VML, and REH designed the laboratory experiments, and analyzed and interpreted the data. JS, SO, GC, PJR, and MC wrote the clinical case series protocol, obtained ethical approval, collated case studies, and interpreted the data. JS, VK, SdB, SO, GC, VM, VML, AM, PJR, JART, BJN, REH, GR, NLB, DES, AC, AN, SY, Y‐JT, AS, FB, and MC participated in the analyses and interpretation of data, wrote or critically reviewed the manuscript, and reviewed and approved the final version.

## Conflict of interest

JS is editor‐in‐chief of Oncogene. JS has sat on a number of scientific advisory boards, including BenevolentAI, and consults with Lansdowne partners and Vitruvian, and since these findings in patients, consults with Eli Lilly and Company; he sits on the Board of Directors for BB Biotech Healthcare Trust. VML is the founder, CEO, and shareholder of HepaPredict AB. In addition, VML discloses consultancy work for EnginZyme AB. VM, AM, SO, MC, and GC report no conflicts of interest. PJR is an employee of BenevolentAI. VK, SdB, JART, BJN, REH, GR, NLB, DES, AN, and AC are employees and shareholders of Eli Lilly and Company.

## Supporting information

Appendix

Review Process File

## Data Availability

Eli Lilly and Company data are available upon request at vivli.org. Access to data is provided after a proposal has been approved by an independent review committee identified for this purpose and after receipt of a signed data‐sharing agreement. Access to data and documents will be provided in a secure data‐sharing environment. For details on submitting a request, see the instructions provided at www.vivli.org. In your request, include the following accession/reference numbers (GenBank accession number MT093571) so that we can identify the related publication and specific dataset that you are trying to access.
