# Supplementary material for: Mechanism of baricitinib supports artificial intelligence‐predicted testing in COVID‐19 patients
Source: EMBO Mol Med. 2020 Jun 24;12(8):e12697. doi: 10.15252/emmm.202012697 (PMC7300657; doi:10.15252/emmm.202012697)
Supplement: Supplementary file 1 — Appendix [file EMMM-12-e12697-s001.pdf]

APPENDIX

Table of Contents

Page 2-3: Appendix **Sacco Baricitinib Study Group**

Page 4-6: Appendix **Table S1.** Detection of SARS-CoV-2 by rRT-PCR in the upper respiratory tract and peripheral blood

Page 7: Appendix **Figure S1.** Classification of COVID-19 disease states and presence of cytokine storm

Page 8: **References**

Sacco Baricitinib Study Group

Hospital name and address:

ASST Fatebenefratelli Sacco

Via Gian Battista Grassi, 74

20157 Milan

Italy

Appendix Sacco Baricitinib Study Group, **in alphabetical order**:

Elena Angeli[1], Spinello Antinori[2,3], Marco Antivalle[4], Barbara Argenteri[1], Davide Bernasconi[1], Anna Maria Brambilla[5], Giacomo Casalini[2,3*], Antonio Castelli[6], Massimo Coen[1], Riccardo Colombo[6], Mario Corbellino[3*], Maria Vittoria Cossu[1], Alice Covizzi[2], Alberto Dolci[2,7], Bramante Augusto Federici[2,8], Massimo Galli[2,3], Maria Rita Gismondo[2,9], Guido Gubertini[1], Alessia Lai[2], Antonella Lattuada[8], Carlo Magni[1], Alessandro Mancon[9], Luca Meroni[3], Laura Milazzo[3], Davide Mileto[9], Fosca Niero[1], Mauro Panteghini[2,7], Marina Petullà[10], Roberto Rech[6], Giuliano Rizzardini[1], Stefano Rusconi[2,3], Piercarlo Sarzi-Puttini[2,4], Marco Schiuma[2], Daniele Scorza[11], Paolo Villa[5], Stefania Vimercati[12]

*denotes authors on the paper

Affiliations:

[1] I Division of Infectious Diseases

[2] "Luigi Sacco" Department of Clinical and Biomedical Sciences, University of Milan

[3] III Division of Infectious Diseases

[4] Division of Rheumatology

[5] Department of Emergency Medicine

[6] Department of General Surgery

[7] Department of Clinical Pathology

[8] Division of Hematology and Transfusion Medicine

[9] Division of Microbiology

[10] Division of Radiology

[11] Division of Nephrology

[12] Division of Pharmacy

All at ASST Fatebenefratelli Sacco, Milan, Italy

Appendix Table S1. Detection of SARS-CoV-2 by rRT-PCR in the upper respiratory tract and peripheral blood

Patient ID	Days post symptom onset	Source	RdRp (Ct value)	N (Ct value)	E (Ct value)
Patient A	4	Nasopharyngeal swab	26	24	24
Patient A	7	Blood	31	31	> 40
Patient A	9	Nasopharyngeal swab	> 40	36	> 40
Patient A	9	Blood	> 40	36	> 40
Patient A	11	Nasopharyngeal swab	> 40	35	> 40
Patient A	11	Blood	> 40	36	> 40
Patient A	14	Nasopharyngeal swab	> 40	37	40
Patient A	14	Blood	> 40	> 40	> 40
Patient A	16	Nasopharyngeal swab	> 40	> 40	> 40
Patient A	16	Blood	> 40	> 40	> 40
Patient A	18	Nasopharyngeal swab	> 40	> 40	> 40
Patient A	21	Nasopharyngeal swab	> 40	34	> 40
Patient A	21	Blood	> 40	38	> 40
Patient A	23	Nasopharyngeal swab	> 40	37	> 40
Patient A	23	Blood	> 40	> 40	> 40
Patient A	25	Nasopharyngeal swab	> 40	37	> 40
Patient A	25	Blood	> 40	> 40	> 40
Patient A	28	Nasopharyngeal swab	> 40	> 40	> 40
Patient A	28	Blood	> 40	> 40	> 40
Patient A	32	Nasopharyngeal swab	> 40	> 40	> 40
Patient A	32	Blood	> 40	> 40	> 40
Patient A	38	Nasopharyngeal swab	> 40	> 40	> 40
Patient A	38	Blood	> 40	> 40	> 40
Patient B	1	Nasopharyngeal swab	24	21	21
Patient B	4	Blood	> 40	> 40	> 40
Patient B	6	Nasopharyngeal swab	31	29	29
Patient B	6	Blood	> 40	> 40	> 40
Patient B	8	Nasopharyngeal swab	32	29	30
Patient B	8	Blood	> 40	> 40	> 40
Patient B	11	Nasopharyngeal swab	36	31	33
Patient B	11	Blood	> 40	> 40	> 40
Patient B	13	Nasopharyngeal swab	> 40	> 40	> 40
Patient B	13	Blood	> 40	> 40	> 40
Patient B	15	Nasopharyngeal swab	> 40	35	> 40
Patient B	15	Blood	> 40	> 40	> 40
Patient B	18	Nasopharyngeal swab	> 40	37	> 40
Patient B	18	Blood	> 40	> 40	> 40
Patient B	20	Nasopharyngeal swab	> 40	> 40	> 40

Patient B	20	Blood	> 40	> 40	> 40
Patient B	22	Nasopharyngeal swab	> 40	38	> 40
Patient B	22	Blood	> 40	> 40	> 40
Patient B	25	Nasopharyngeal swab	> 40	> 40	> 40
Patient B	25	Blood	> 40	> 40	> 40
Patient B	27	Nasopharyngeal swab	> 40	> 40	> 40
Patient B	27	Blood	> 40	> 40	> 40
Patient B	34	Nasopharyngeal swab	> 40	> 40	> 40
Patient B	34	Blood	> 40	> 40	> 40
Patient B	36	Blood	> 40	> 40	> 40
Patient C	8	Nasopharyngeal swab	35	32	33
Patient C	9	Blood	> 40	37	> 40
Patient C	11	Nasopharyngeal swab	> 40	35	38
Patient C	11	Blood	> 40	38	> 40
Patient C	13	Nasopharyngeal swab	34	29	30
Patient C	13	Blood	> 40	37	> 40
Patient C	16	Nasopharyngeal swab	26	23	23
Patient C	16	Blood	> 40	38	> 40
Patient C	18	Nasopharyngeal swab	> 40	> 40	> 40
Patient C	18	Blood	> 40	37	> 40
Patient C	20	Nasopharyngeal swab	> 40	> 40	> 40
Patient C	20	Blood	> 40	> 40	> 40
Patient C	23	Nasopharyngeal swab	> 40	> 40	> 40
Patient C	23	Blood	> 40	> 40	> 40
Patient C	25	Nasopharyngeal swab	> 40	> 40	> 40
Patient C	25	Blood	> 40	> 40	> 40
Patient C	27	Nasopharyngeal swab	> 40	> 40	> 40
Patient C	27	Blood	> 40	> 40	> 40
Patient C	30	Nasopharyngeal swab	> 40	> 40	> 40
Patient C	30	Blood	> 40	> 40	> 40
Patient C	34	Blood	> 40	> 40	> 40
Patient C	41	Blood	> 40	> 40	> 40
Patient D	7	Nasopharyngeal swab	> 40	31	38
Patient D	8	Nasopharyngeal swab	> 40	32	34
Patient D	9	Blood	> 40	> 40	> 40
Patient D	11	Nasopharyngeal swab	> 40	35	> 40
Patient D	11	Blood	> 40	> 40	> 40
Patient D	14	Nasopharyngeal swab	> 40	36	> 40
Patient D	14	Blood	> 40	38	> 40
Patient D	16	Nasopharyngeal swab	> 40	> 40	> 40
Patient D	16	Blood	> 40	> 40	> 40
Patient D	18	Nasopharyngeal swab	> 40	> 40	> 40

Patient D	18	Blood	> 40	> 40	> 40
Patient D	21	Nasopharyngeal swab	> 40	> 40	> 40
Patient D	21	Blood	> 40	> 40	> 40
Patient D	23	Nasopharyngeal swab	> 40	> 40	> 40
Patient D	23	Blood	> 40	> 40	> 40
Patient D	25	Blood	> 40	> 40	> 40
Patient D	30	Nasopharyngeal swab	> 40	> 40	> 40
Patient D	30	Blood	> 40	> 40	> 40
Patient D	32	Blood	> 40	> 40	> 40

Three viral target genes, RNA-dependent RNA polymerase (RdRp), nucleocapsid protein (N) and envelope membrane (E) (Corman, Landt et al., 2020), together with the housekeeping gene GAPDH were simultaneously amplified. Negative results for SARS-CoV-2 detection were defined as those with Ct values > 40. This is a stringent cut-off compared to other studies (Gautret, Lagier et al., 2020). In addition, the most sensitive target gene (N gene) was chosen for detection. Note on discharge criteria: all patients have been discharged after at least 2 consecutive negative NPSs with the exception of patient A. Patient A was discharged after only one negative NPS. This was because she was clinically improved and fully able to quarantine at home with her husband in accordance with WHO guidelines. On 4/17/2020 (39 days post-symptom onset) her husband tested negative on both NPS rRT-PCR and SARS-CoV-2 serology. It is relevant to note that capacity pressures on the hospital were overwhelming. Discharge criteria suggested by the regional health authority have changed during the epidemics considering a case-by-case basis. We monitored Patient A, a nurse, closely: the NPS was repeated the day after discharge (day 18 after symptom onset) and no virus was amplified. She returned to work in Milan on 4/21/2020

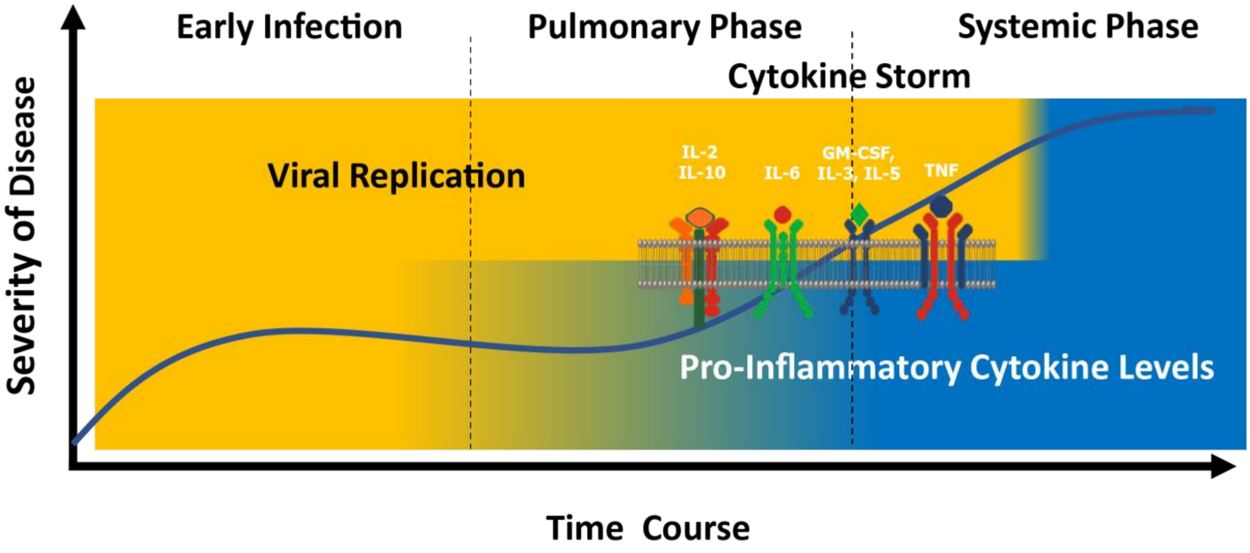

Appendix Figure S1. Classification of COVID-19 disease states and presence of cytokine storm

Escalating phases of disease progression with COVID-19, with associated signs, symptoms, and presence of cytokine storm are shown. Baricitinib is a potent inhibitor of several cytokines implicated in COVID-19 (except TNF shown in figure). *Adapted from Siddiqi, et al (Siddiqi & Mehra, 2020).

References

Corman VM, Landt O, Kaiser M, Molenkamp R, Meijer A, Chu DKW, Bleicker T, Brunink S, Schneider J, Schmidt ML, Mulders D, Haagmans BL, van der Veer B, van den Brink S, Wijsman L, Goderski G, Romette JL, Ellis J, Zambon M, Peiris M et al. (2020) Detection of 2019 novel coronavirus (2019-nCoV) by real-time RT-PCR. *Euro Surveill* 25

Gautret P, Lagier JC, Parola P, Hoang VT, Meddeb L, Mailhe M, Doudier B, Courjon J, Giordanengo V, Vieira VE, Dupont HT, Honore S, Colson P, Chabriere E, La Scola B, Rolain JM, Brouqui P, Raoult D (2020) Hydroxychloroquine and azithromycin as a treatment of COVID-19: results of an open-label non-randomized clinical trial. *Int J Antimicrob Agents*: 105949

Siddiqi HK, Mehra MR (2020) COVID-19 Illness in Native and Immunosuppressed States: A Clinical-Therapeutic Staging Proposal. *The Journal of Heart and Lung Transplantation*
