# Supplementary material for: Mechanism of baricitinib supports artificial intelligence‐predicted testing in COVID‐19 patients
Source: EMBO Mol Med. 2020 Jun 24;12(8):e12697. doi: 10.15252/emmm.202012697 (PMC7300657; doi:10.15252/emmm.202012697)
Supplement: Supplementary file 2 — Review Process File [file EMMM-12-e12697-s002.pdf]

EMBO Molecular Medicine

Mechanism of baricitinib supports artificial intelligence-predicted testing in COVID-19 patients

Venkatesh Krishnan, Justin Stebbing, Stephanie de Bono, Silvia Ottaviani, Giacomo Casalini, Peter Richardson, Vanessa Monteil, Volker Lauschke, Ali Mirazimi, Sonia Youhanna, Yee-Joo Tan, Fausto Baldanti, Antonella Sarasini, Jorge Alfonso Terres, Brian Nickoloff, Richard Higgs, Guilherme Rocha, Nicole Byers, Douglas Schlichting, Ajay Nirula, Anabela Cardoso, and Mario Corbellino
DOI: 10.15252/emmm.202012697

Corresponding author(s): Venkatesh Krishnan (KRISHNAN_GARY@LILLY.COM)

Review Timeline:

Submission Date:	16th May 20
Editorial Decision:	22nd May 20
Revision Received:	22nd May 20
Accepted:	28th May 20

Editor: Celine Carret

Transaction Report:

(Please note that the manuscript was transferred from another journal where it was originally reviewed. Since the original reviews are not subject to EMBO's transparent review process policy, the reports and author response cannot be published.)

EMBO PRESS

YOU MUST COMPLETE ALL CELLS WITH A PINK BACKGROUND ➔

PLEASE NOTE THAT THIS CHECKLIST WILL BE PUBLISHED ALONGSIDE YOUR PAPER

Corresponding Author Name:Venkatesh Krishnan

Journal Submitted to: EMBO Molecular Medicine

Manuscript Number: EMM-2020-12697

Reporting Checklist For Life Sciences Articles (Rev. June 2017)

This checklist is used to ensure good reporting standards and to improve the reproducibility of published results. These guidelines are consistent with the Principles and Guidelines for Reporting Preclinical Research issued by the NIH in 2014. Please follow the journal's authorship guidelines in preparing your manuscript.

A- Figures

1. Data

The data shown in figures should satisfy the following conditions:

- the data were obtained and processed according to the field's best practice and are presented to reflect the results of the experiments in an accurate and unbiased manner.
- figure panels include only data points, measurements or observations that can be compared to each other in a scientifically meaningful way.
- graphs include clearly labeled error bars for independent experiments and sample sizes. Unless justified, error bars should not be shown for technical replicates.
- if n< 5, the individual data points from each experiment should be plotted and any statistical test employed should be justified
- Source Data should be included to report the data underlying graphs. Please follow the guidelines set out in the author ship guidelines on Data Presentation.

2. Captions

Each figure caption should contain the following information, for each panel where they are relevant:

- a specification of the experimental system investigated (eg cell line, species name).
- the assay(s) and method(s) used to carry out the reported observations and measurements
- an explicit mention of the biological and chemical entity(ies) that are being measured.
- an explicit mention of the biological and chemical entity(ies) that are altered/varied/perturbed in a controlled manner.
- the exact sample size (n) for each experimental group/condition, given as a number, not a range;
- a description of the sample collection allowing the reader to understand whether the samples represent technical or biological replicates (including how many animals, litters, cultures, etc.).
- a statement of how many times the experiment shown was independently replicated in the laboratory.
- definitions of statistical methods and measures:
  - common tests, such as t-test (please specify whether paired vs. unpaired), simple χ^2 tests, Wilcoxon and Mann-Whitney tests, can be unambiguously identified by name only, but more complex techniques should be described in the methods section;
  - are tests one-sided or two-sided?
  - are there adjustments for multiple comparisons?
  - exact statistical test results, e.g., P values = x but not P values < x;
  - definition of 'center values' as median or average;
  - definition of error bars as s.d. or s.e.m.

Any descriptions too long for the figure legend should be included in the methods section and/or with the source data.

In the pink boxes below, please ensure that the answers to the following questions are reported in the manuscript itself. Every question should be answered. If the question is not relevant to your research, please write NA (non applicable). We encourage you to include a specific subsection in the methods section for statistics, reagents, animal models and human subjects.

USEFUL LINKS FOR COMPLETING THIS FORM

http://www.antibodypedia.com
http://1degreebio.org
http://www.equator-network.org/reporting-guidelines/improving-bioscience-research-repor
http://grants.nih.gov/grants/olaw/olaw.htm
http://www.mrc.ac.uk/Ourresearch/Ethicsresearchguidance/Useofanimals/index.htm
http://ClinicalTrials.gov
http://www.consort-statement.org
http://www.consort-statement.org/checklists/view/32-consort/66-title
http://www.equator-network.org/reporting-guidelines/reporting-recommendations-for-tumo
http://datadryad.org
http://figshare.com
http://www.ncbi.nlm.nih.gov/gap
http://www.ebi.ac.uk/ega
http://biomodels.net/
http://biomodels.net/miriam/
http://jjj.biochem.sun.ac.za
http://oba.od.nih.gov/biosecurity/biosecurity_documents.html
http://www.selectagents.gov/

B- Statistics and general methods

Please fill out these boxes ➔ (Do not worry if you cannot see all your text once you press return)

1.a. How was the sample size chosen to ensure adequate power to detect a pre-specified effect size?	For NCT01469013, this information is described in the primary manuscript (Tanaka et al. 2016. J Rheumatol 43: 504-11).
1.b. For animal studies, include a statement about sample size estimate even if no statistical methods were used.	N/A
2. Describe inclusion/exclusion criteria if samples or animals were excluded from the analysis. Were the criteria pre-established?	For NCT01469013, this information is described in the primary manuscript (Tanaka et al. 2016. J Rheumatol 43: 504-11). For the clinical case series, inclusion criteria for compassionate use of an investigational oral medicine (albeit one that was approved in another indication) for patients with signs of severe illness at diagnosis or secondary clinical aggravation (respiratory symptoms or general signs) was based on World Health Organization (WHO) criteria for severe pneumonia caused by SARS-CoV-2 (WHO 2020).
3. Were any steps taken to minimize the effects of subjective bias when allocating animals/samples to treatment (e.g. randomization procedure)? If yes, please describe.	NCT01469013 was a double-blind, randomized, placebo-controlled, phase 2b study (Tanaka et al. 2016. J Rheumatol 43: 504-11). The clinical case series involved compassionate use of the study drug.
For animal studies, include a statement about randomization even if no randomization was used.	N/A
4.a. Were any steps taken to minimize the effects of subjective bias during group allocation or/and when assessing results (e.g. blinding of the investigator)? If yes please describe.	NCT01469013 was a double-blind, randomized, placebo-controlled, phase 2b study (Tanaka et al. 2016. J Rheumatol 43: 504-11). The clinical case series involved compassionate use of the study drug.
4.b. For animal studies, include a statement about blinding even if no blinding was done	N/A
5. For every figure, are statistical tests justified as appropriate?	Yes this has been justified as appropriate as per standard statistical reporting for papers of this kind, with the statistical test specified. Standard statistical methods for fitting concentration response curves and longitudinal data analysis (mixed effect models) were utilized.
Do the data meet the assumptions of the tests (e.g., normal distribution)? Describe any methods used to assess it.	Yes this has been justified as appropriate as per standard statistical reporting for papers of this kind, with the statistical test specified. Analyte concentration data (e.g. IL-6 and pSTATs) are frequently non-Normal and appropriate log transformations were used prior to statistical analysis.
Is there an estimate of variation within each group of data?	As above. All statistical results are reported with testing results (which are based on appropriate residual variance estimates) and standard errors are shown where applicable.
Is the variance similar between the groups that are being statistically compared?	As above. Yes, this is one of the main reasons for using log transformed data as noted above.

C- Reagents

6. To show that antibodies were profiled for use in the system under study (assay and species), provide a citation, catalog number and/or clone number, supplementary information or reference to an antibody validation profile. e.g., Antibodypedia (see link list at top right), 1DegreeBio (see link list at top right).	The standard clinical tests and antibodies have been specified when appropriate with company names/locations, when appropriate for standard reporting in scientific manuscripts. Detection of IgG antibodies against the S1/S2 antigens of SARS-CoV-2 using the LIAISON SARS-CoV-2 S1/S2 IgG Kit (DiaSorin S.p.A., Saluggia, Italy). The monoclonal primary antibody anti-1A9 targeted against SARS-CoV-2 (diluted in PBTA to a final concentration 5µg/ml).
7. Identify the source of cell lines and report if they were recently authenticated (e.g., by STR profiling) and tested for mycoplasma contamination.	The use of cell lines is described and referenced within the paper appropriately, as per standard reporting in scientific manuscripts.

* for all hyperlinks, please see the table at the top right of the document

D- Animal Models

8. Report species, strain, gender, age of animals and genetic modification status where applicable. Please detail housing and husbandry conditions and the source of animals.	N/A
9. For experiments involving live vertebrates, include a statement of compliance with ethical regulations and identify the committee(s) approving the experiments.	N/A
10. We recommend consulting the ARRIVE guidelines (see link list at top right) (PLoS Biol. 8(6), e1000412, 2010) to ensure that other relevant aspects of animal studies are adequately reported. See author guidelines, under 'Reporting Guidelines'. See also: NIH (see link list at top right) and MRC (see link list at top right) recommendations. Please confirm compliance.	N/A

E- Human Subjects

11. Identify the committee(s) approving the study protocol.	For NCT01469013, all investigation sites received approval from the appropriate authorized institutional review board or ethics committee. For the clinical case sereies, ethics and local Institutional Review Board approval was authorized on March 16, 2020 (number 14581/2020), which provided permission to treat three patients with bilateral COVID-19 pneumonia with baricitinib. A supplementary approval was sought on March 25, 2020 and obtained to treat a fourth patient for validation purposes.
12. Include a statement confirming that informed consent was obtained from all subjects and that the experiments conformed to the principles set out in the WMA Declaration of Helsinki and the Department of Health and Human Services Belmont Report.	NCT01469013 was conducted in accordance with ethical principles of the Declaration of Helsinki and Good Clinical Practice guidelines. All investigation sites received approval from the appropriate authorized institutional review board or ethics committee. All patients provided written informed consent before the study-related procedures were undertaken. Whole blood samples from healthy volunteers were obtained under a protocol approved by the Institutional Review Board of Stanford University (reference numbers eProtocol # 38735 and IRB Registration # 6208). The IRB provided a Waiver of Consent because leukocyte-enriched fractions are by-products of the blood donation.
13. For publication of patient photos, include a statement confirming that consent to publish was obtained.	N/A
14. Report any restrictions on the availability (and/or on the use) of human data or samples.	Eli Lilly and Company data are available upon request at vivli.org. Access to data is provided after a proposal has been approved by an independent review committee identified for this purpose and after receipt of a signed data sharing agreement. Access to data and documents will be provided in a secure data sharing environment. For details on submitting a request, see the instructions provided at www.vivli.org. In your request, please include the following accession/reference numbers so that we can identify the related publication and specific dataset that you're trying to access.
15. Report the clinical trial registration number (at ClinicalTrials.gov or equivalent), where applicable.	NCT01469013
16. For phase II and III randomized controlled trials, please refer to the CONSORT flow diagram (see link list at top right) and submit the CONSORT checklist (see link list at top right) with your submission. See author guidelines, under 'Reporting Guidelines'. Please confirm you have submitted this list.	N/A (primary data for NCT01469013 are published in Tanaka et al. 2016. J Rheumatol 43: 504-11)
17. For tumor marker prognostic studies, we recommend that you follow the REMARK reporting guidelines (see link list at top right). See author guidelines, under 'Reporting Guidelines'. Please confirm you have followed these guidelines.	N/A

F- Data Accessibility

18: Provide a "Data Availability" section at the end of the Materials & Methods, listing the accession codes for data generated in this study and deposited in a public database (e.g. RNA-Seq data: Gene Expression Omnibus GSE39462, Proteomics data: PRIDE PXD000208 etc.) Please refer to our author guidelines for 'Data Deposition'.<br><br>Data deposition in a public repository is mandatory for:<br>a. Protein, DNA and RNA sequences<br>b. Macromolecular structures<br>c. Crystallographic data for small molecules<br>d. Functional genomics data<br>e. Proteomics and molecular interactions	Eli Lilly and Company data are available upon request at vivli.org. Access to data is provided after a proposal has been approved by an independent review committee identified for this purpose and after receipt of a signed data sharing agreement. Access to data and documents will be provided in a secure data sharing environment. For details on submitting a request, see the instructions provided at www.vivli.org. In your request, please include the following accession/reference numbers (Genbank accession number MT093571) so that we can identify the related publication and specific dataset that you're trying to access.
19. Deposition is strongly recommended for any datasets that are central and integral to the study; please consider the journal's data policy. If no structured public repository exists for a given data type, we encourage the provision of datasets in the manuscript as a Supplementary Document (see author guidelines under 'Expanded View' or in unstructured repositories such as Dryad (see link list at top right) or Figshare (see link list at top right).	N/A
20. Access to human clinical and genomic datasets should be provided with as few restrictions as possible while respecting ethical obligations to the patients and relevant medical and legal issues. If practically possible and compatible with the individual consent agreement used in the study, such data should be deposited in one of the major public access-controlled repositories such as dbGAP (see link list at top right) or EGA (see link list at top right).	N/A
21. Computational models that are central and integral to a study should be shared without restrictions and provided in a machine-readable form. The relevant accession numbers or links should be provided. When possible, standardized format (SBML, CellML) should be used instead of scripts (e.g. MATLAB). Authors are strongly encouraged to follow the MIRIAM guidelines (see link list at top right) and deposit their model in a public database such as Biomodels (see link list at top right) or JWS Online (see link list at top right). If computer source code is provided with the paper, it should be deposited in a public repository or included in supplementary information.	N/A

G- Dual use research of concern

22. Could your study fall under dual use research restrictions? Please check biosecurity documents (see link list at top right) and list of select agents and toxins (APHIS/CDC) (see link list at top right). According to our biosecurity guidelines, provide a statement only if it could.	No
